# High Variation of Fluorescence Protein Maturation Times in Closely Related *Escherichia coli* Strains

**DOI:** 10.1371/journal.pone.0075991

**Published:** 2013-10-14

**Authors:** Elke Hebisch, Johannes Knebel, Janek Landsberg, Erwin Frey, Madeleine Leisner

**Affiliations:** 1 Department of NanoBiophotonics, Max-Planck-Institute for Biophysical Chemistry, Göttingen, Germany; 2 Arnold-Sommerfeld-Center for Theoretical Physics and Center for NanoScience, Department of Physics, Ludwig-Maximilians Universität München, München, Germany; 3 Laboratoire Interdisciplinaire de Physique, Universite Joseph Fourier de Grenoble, Saint Martin d'Heres, France; Griffith University, Australia

## Abstract

Fluorescent proteins (FPs) are widely used in biochemistry, biology and biophysics. For quantitative analysis of gene expression FPs are often used as marking molecules. Therefore, sufficient knowledge of maturation times and their affecting factors is of high interest. Here, we investigate the maturation process of the FPs GFP and mCherry expressed by the three closely related *Escherichia coli* strains of the Colicin E2 system, a model system for colicinogenic interaction. One strain, the C strain produces Colicin, a toxin to which the S strain is sensitive, and against which the R strain is resistant. Under the growth conditions used in this study, the S and R strain have similar growth rates, as opposed to the C strain whose growth rate is significantly reduced due to the toxin production. In combination with theoretical modelling we studied the maturation kinetics of the two FPs in these strains and could confirm an exponential and sigmoidal maturation kinetic for GFP and mCherry, respectively. Our subsequent quantitative experimental analysis revealed a high variance in maturation times independent of the strain studied. In addition, we determined strain dependent maturation times and maturation behaviour. Firstly, FPs expressed by the S and R strain mature on similar average time-scales as opposed to FPs expressed by the C strain. Secondly, dependencies of maturation time with growth conditions are most pronounced in the GFP expressing C strain: Doubling the growth rate of this C strain results in an increased maturation time by a factor of 1.4. As maturation times can vary even between closely related strains, our data emphasize the importance of profound knowledge of individual strains' maturation times for accurate interpretation of gene expression data.

## Introduction

Bacterial competition and interaction [1] influence the spreading of bacterial infections [2], and have been studied both experimentally [3]–[6] and theoretically [6]–[9]. Under laboratory conditions, competition of species is explored under well-defined conditions, with strains similar in growth rate and competition taking place in the exponential growth phase of each particular contestant. Natural competition, however, is not restricted to a particular growth phase and becomes strongest in the stationary growth phase when resources are limited. In addition, complex systems such as biofilms are composed of different species [10], [11] that are strongly varying in their respective growth rates or fitnesses. Such unbalanced growth conditions, which are present in natural habitats, highly affect ongoing multi-species interactions because species in different growth phases interact with each other. Multi-species competition is often monitored by using fluorescent proteins (FPs), which are expressed in addition to the normal metabolism, for each competitor. Since gene expression is directly coupled to lag-time (the time after which a population has overcome the lag-phase) and growth rate [12] and therewith to the growth conditions of a particular species, two questions immediately arise: Do the growth conditions affect the expression of the fluorescent markers? And how reliable can we monitor bacterial interaction dynamics by using fluorescent markers expressed by the studied organisms?

Expression of fluorescent proteins involves transcription and translation. Post-translation, the chromophore of the fluorescent protein is formed resulting in the fluorescent state of the FP. The time needed to form the FPs' chromophore is defined as the maturation time (MT) [13]. The best-known representative of the FPs' family is the green fluorescent protein (GFP), isolated from the jellyfish *Aequorea victoria* [14], [15]. Genetic engineering allowed for optimization of GFP characteristics such as higher quantum yield, increased photo-stability or shorter maturation times. By now, also a large toolbox of differently colored variants of FPs is available [16]–[19]. Depending on the experimental question addressed, the choice of the fluorescent protein is crucial: Whereas for protein localization strong fluorescence is a prerequisite to visualize even tiny amounts of the protein of interest [20], [21], experiments involving time-lapse microscopy require short maturation times. The properties of the chosen FPs are even more important as soon as quantitative analysis of bacterial interaction dynamics is performed at the single cell level and the obtained data are directly incorporated into theoretical models. In this case, the maturation time of the fluorescent protein is a limiting factor for the experimental time resolution and has to be taken into account for data analysis. For GFP the post-translational maturation takes place without the assistance of external enzymes or cofactors except for molecular oxygen (O_2_) [22]–[24]. The GFP maturation involves a series of chemical modifications of the GFP's chromophore [22], [23], the slowest of them by far being an oxidation step. Therefore, GFP maturation can be described as a one-step process [25]–[27]. Other FPs such as DsRed that was isolated from *Discosoma sp*. [28] mature in two subsequent oxidation steps [27], [29]. As a consequence, maturation times are highly variable for different fluorescent proteins [13]. It was shown that external factors such as temperature [23], [30], pH value [29], [31], or the bacterial species chosen to express the fluorescent protein [13], [32] can affect the maturation time. Although interactions of closely related organisms are widely studied (e.g., the occurrence of cheaters in a population of public good producers [33], [34]), only little is known about the variation of maturation times of fluorescent proteins that are expressed in closely related bacterial strains of the same species (e.g., wild-type versus mutant strains).

In this study, we quantitatively investigated the maturation times of GFPmut3 [30] (in the following named GFP) and mCherry (mCh) [35], a derivative of DsRed [18] for the *E. coli* wild-type strain BZB 1011, and two genetically different derivatives of BZB 1011. These strains represent a well-studied bacterial model system of colicinogenic interaction [6], [7], [36], [37]. While two strains have comparable growth rates, the third and Colicin producing strain has a considerably reduced growth rate. We used this system to study whether growth conditions as reflected by growth rate and lag-time can in general affect the maturation process of FPs. We further investigated the dependence of maturation on the fluorescent protein, and asked whether there are strain specific effects that lead to an increase or decrease of maturation times. In a combined experimental and theoretical approach we investigated the kinetic mechanisms of GFP and mCherry maturation. We report a high variance of maturation times of FPs even when expressed within one strain. Maturation profiles (correlations of MT versus growth rate, lag-time, or maximal fluorescence intensity (FI)) were different for different strains indicating that maturation is affected by the strains' different metabolisms.

## Materials and Methods

### Strains and growth conditions

Strains used in this study represent the *E. coli* Colicin E2 system (BZB1011 (S), E2^C^-BZB1011 (C) and E2^R^-BZB1011 (R)) as described in [6]. To study maturation times of fluorescent proteins, plasmids expressing either the green fluorescent protein GFPmut3 (GFP [30]) or mCherry (mCh [35]) from the inducible pBAD promoter, have been introduced into the three different strains. Bacteria were grown in liquid M63 medium in the case of overnight cultures at 37°C supplemented with glycerol (0.2%) and Casamino acid (0.2%). We added arabinose (0.2%) for maximal fluorescence induction and ampicillin (100 µg/ml) for plasmid maintenance.

### Plasmids used in this study

All fluorescent proteins are expressed from the arabinose inducible promoter pBAD as present in the plasmid pBAD24. Introduction of the fluorescent proteins resulted in the plasmids pBAD24-GFP [38] and pBAD24-mcherry (this study). Plasmid pBAD24-mCherry was created by exchanging the GFP in pBAD24-GFP by using EcoRI and HindIII restriction sites. mCherry was obtained via PCR from the pBRcherry [35] by using the primer pair P_forward_: 5′-tggccagaattccccggg-3′ and P_reverse_: 5′-catgtttgacagcttatcatcgataagctt-3′. To prevent plasmid-loss, both plasmids carry an ampicillin antibiotic resistance.

### Determination of absorbance and fluorescence intensities

Strains were grown in 0.5 ml of M63 liquid medium in a 48 well plate with initial optical density (OD) 0.1. OD at 600 nm was obtained every 15 min over a time period of 680 min at 37°C, shaking at 300 rpm in a microplate reader (BMG Labtech) (Figure S1). In parallel, the temporal development of fluorescence intensity for GFP and mCherry was measured. The filtersets used for this purpose were: GFP excitation at 485 nm, GFP emission at 520 nm, mCherry excitation at 584 nm and mCherry emission at 620 nm.

### Analysis of growth rates (GR) and lag-time (LT)

#### Growth rate

After blank correction, the growth rate was obtained as follows: Growth curves as represented by OD 600 nm were fitted by using the sigmoidal fit function f_S_ of the IGOR PRO 4.06 software. The natural logarithm of this fit function was taken, yielding the function f_N_ = ln(f_S_(t)). Since the exponential growth phase corresponds to the domain of the steepest slope of the function f_N_(t), we obtain maximum exponential growth by differentiating f_N_(t) with respect to the time t and determining its maximum value: b = max((d/dt) f_N_(t)). The growth rate (GR) is then calculated according to: GR = b/ln(2).

#### Lag-time

The lag-phase represents the period of time a bacterial population needs to adjust to new environmental conditions. In our experiments it is the time the bacterial culture needs to start growing again after transfer from the overnight culture into new fresh medium. Therefore, the lag-time denotes the beginning of the exponential growth phase and corresponds to the first inflection point of the first derivative of the function f_N_(t). It was thus obtained by extracting the time corresponding to the maximum of the second derivative of f_N_(t) with respect to t. The fit protocols for both the growth rate and the lag-times were performed at 5% significance level.

### Determination of maturation times (MT)

**Figure 1:**
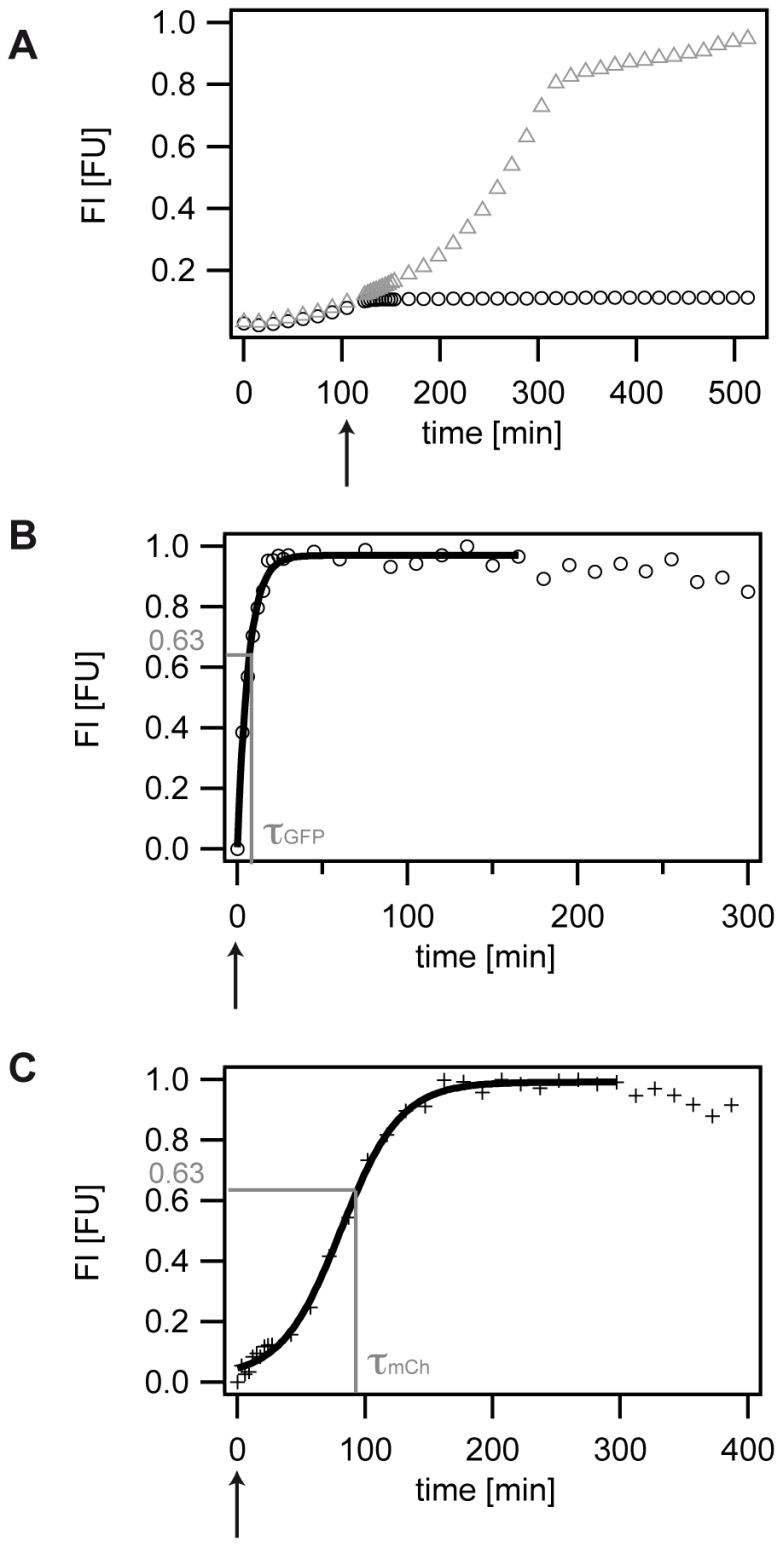
Analyzing maturation times (MT). A) Typical data set of fluorescence development of the S strain expressing GFP in the absence (grey triangles) and presence (black circles) of 200 µg/ml CAP. An arrow indicates the addition of CAP after 120 min. B) Typical data set of fluorescence development of the S strain expressing GFP in the presence of 200 µg/ml CAP. The arrow indicates the addition of CAP. Fluorescence development is fitted with an exponential fit, yielding τ_GFP_ at normalized 63% FI. The maturation time then corresponds to the inverse of the characteristic time constant τ_GFP_ of the exponential fit. C) Typical data set of fluorescence development of the S strain expressing mCh in the presence of 200 µg/ml CAP. The arrow indicates the addition of CAP. Fluorescence development is fitted with a sigmoidal fit, yielding τ_mCh_ at normalized 63% FI. The maturation time then corresponds to the inverse of the characteristic time constant τ_mCh_ of the sigmoidal fit.

Bacterial cells were grown as described above in a 48-well microtiter plate, and optical density and fluorescence development was recorded by using a microplate reader (BMG Labtech). To analyze maturation times, cells were grown until all three strains had clearly entered the exponential growth phase. This allowed for maximum fluorescence expression and ensured that all strains are in the same population cell phase, namely the exponential growth phase to directly compare the obtained data. Since mCherry expressing strains grow considerably slower than GFP expressing strains (Table S1), they reach the mid-exponential phase later than the GFP expressing strains. Therefore, we added the antibiotic chloramphenicol at different time points in experiments investigating GFP or mCherry maturation, namely at 120 min (GFP) and 180 min (mCherry) (Figure 1). CAP inhibits translation of the gram-negative *Escherichia coli* strains [39]. Consequently, all fluorescence arising after addition of CAP is due to post-translational maturation of the fluorescent protein. To ensure complete translation inhibition, CAP was added at 200 µg/ml, a concentration that significantly exceeds the minimal inhibitory concentration (MIC) of 20–40 µg/ml as stated in literature for *E. coli* [40]. After CAP addition, OD and fluorescence measurement intervals were reduced to 3 min for 30 min for better time-resolution and eventually set to 15 min again for the rest of the experiment.

### Calculation of maturation times (MT)

For both the GFP and the mCherry fluorescence data, the time-point of CAP addition and the corresponding basal value of FI were set to 0. FI data were fitted in the time window between the addition of CAP and the saturation of the FI signal. The saturation value was defined as the average value of data points before a drop in signal by over 5% set in due to cell lysis. This way, miscalculations of the MT according to possible degradation of the FPs could be ruled out.

#### Calculation of GFP maturation time

After a blank correction, the GFP fluorescence data were fitted by using the exponential fit function f_e_ of the IGOR PRO 4.06 software. The MT was determined as the characteristic time constant of the exponential fit, denoting the point in time at which about 63% of the saturated fluorescence intensity was reached.

#### Calculation of mCh maturation time

After a blank correction, the mCh fluorescence data were fitted using the sigmoidal fit function f_S_ of the IGOR PRO 4.06 software. The 63% value of the fit's FI saturation value was determined and the corresponding MT was extracted as the mCh's maturation time.

The fit protocols for both the growth rate and the lag-times were performed at 5% significance level.

## Results

### The *E. coli* Colicin E2 system

In the present study we quantitatively analyze the maturation behavior of two different FPs that are expressed by three distinct *Escherichia coli* strains of the well-characterized *E. coli* Colicin E2 System [6], [41]. We chose this bacterial system because it represents a model system to study colicinogenic bacterial interactions. This system is comprised of a strain that produces the toxin Colicin E2 (C), a strain that is sensitive to Colicin (S), and a strain that is resistant to the Colicin (R). Since Colicin production bears some metabolic costs, the C strain grows significantly slower than the S and R strain whose growth rates are similar under the growth conditions used in this study (Figure S1A, Table S1). The strains' genetic differences in combination with the observed growth rate differences make this bacterial model system an ideal candidate to address the question whether maturation times of FPs differ for closely related bacteria. To this end, we investigated the maturation of the fluorescent protein GFPmut3 (GFP) and the red fluorescent protein mCherry. Both FPs are commonly used for pairwise gene expression studies [31], [42], and differ in their chromophore formation processes [25]–[27], [29] and maturation times [13], [18], [19].

To study maturation of the fluorescent proteins GFP (Figure 1A,B) and mCherry (Figure 1C), plasmids expressing the fluorescent proteins from the arabinose inducible promoter pBAD were transferred into the three strains (see Material and Methods). While expression of GFP in the S, R, and C strain caused only a small growth rate reduction as compared to the non-fluorescent strains, the growth rate was significantly reduced in strains expressing mCherry (Table S1, Figure S1). This finding is in accordance with previous studies showing that additionally expressed proteins can reduce growth rates [12]. We observed that the shift towards reduced growth rates was comparable for all three strains. The ratio of growth rates between the S, R, and C strain was sustained, leaving the C strain the bacterium with the lowest growth rate as compared to the S and R strain (Table S1).

### High variation of maturation times

Growth conditions can alter the metabolism of organisms and can best be analyzed by measuring the parameters growth rate and lag-time. To address the impact of growth conditions as represented by the growth rate and the lag-time of the population, we experimentally induced day-to-day variations in these parameters by diluting overnight cultures only to an optical density of 0.1 (1:10 dilution) [43]. Table S1 gives the growth rates of the whole population for the particular experiment shown in Figure S1. The errors given in this table correspond to three different replicas (wells of the microtiter plate). Whereas well-to-well variations of experiments performed on one day are quite small, the day-to-day variations in the populations' overall growth rates and lag-times are considerably higher (Table S2). This way, we were able to investigate maturation times of mCherry or GFP in dependence on growth rate or lag-time variations.

As a first attempt, we analyzed the maturation time of GFP for the S strain using a well-established method [32], [38]. Bacterial cells were grown to exponential growth phase to ensure maximum production of the fluorescent protein and to minimize any cell stage specific effects on the maturation time of the FPs. To further compare the maturation of FPs expressed by the different strains, 200 µg/ml of the antibiotic chloramphenicol (CAP) were added at the same time-point, namely 120 min (Figure 1A,B). In the absence of CAP, cells grew continuously until stationary growth phase and reached high fluorescence values (Figure 1A). In the presence of CAP, growth ceased after CAP addition and only a small, but significantly detectable further increase in fluorescence intensity could be observed. CAP arrests protein production by inhibiting the enzyme Peptidyltransferase [39], an essential part of the ribosome being responsible for translation. Therefore, any FI signal arising after CAP addition can be attributed to chromophore maturation of the fluorescent protein. Fitting to this data of saturating fluorescence development yields the maturation time of the fluorescent protein expressed in a particular bacterial strain (Material and Methods). For a quantitative analysis of maturation times as presented in the following, complete translation inhibition and a high stability of the fluorescent proteins are prerequisites. To ensure complete translation inhibition, CAP was added at a concentration significantly above the minimal inhibitory concentration (MIC) [40]. In addition, we performed control experiments verifying that no strain-specific effects might interfere with translation inhibition via CAP (Figure S2, Table S3). Furthermore, the fluorescent proteins GFPmut3 and mCherry are known to be very stable [18], [44], [45], with a half-life time of GFPmut3 with 24 hours in *E. coli* [45]. To rule out that these FPs might be less stable in the three specific strains used in this study, we monitored the fluorescence intensity after CAP addition for several hours and found both FPs to be stable within the investigated time frame in all three strains (Figure S3).

GFP maturation can be described theoretically as a one-step process [25]–[27]. We verified this model for our experimental data (Supporting Information S1) and subsequently applied an exponential fit to the obtained experimental data (Material and Methods). We determined an average maturation time of 5.38±0.2 min at normalized fluorescence intensity, FI = 63% for the S strain (Figure 1A, Table S2) at an average growth rate of 0.75±0.08 1/h and lag-time 52.5±8.3 min. This value is in accordance to other maturation times for GFP obtained in *E. coli* strains [8], [14], validating the suitability of our approach.

**Figure 2:**
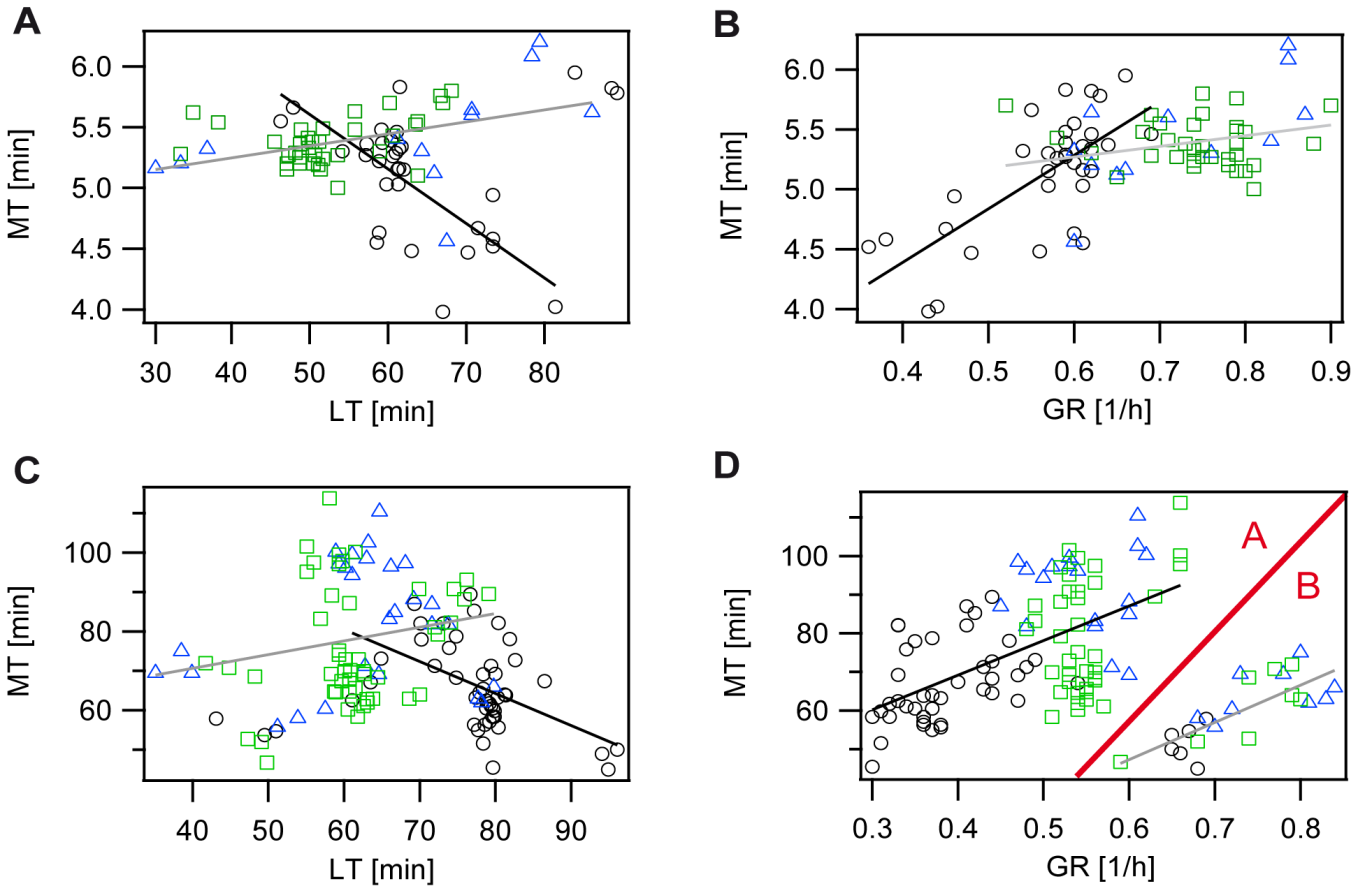
Analysis of maturation time (MT) in dependence on growth conditions (lag-time (LT) and growth rate (GR)) at 200 µg/ml CAP for the S (green rectangles), R (blue triangles), and C strain (black circles). Solid lines: fits to experimental data. Fit-parameter and Pearson's correlation coefficients can be found in Table S4. A) and B) strains expressing GFP, C) and D) strains expressing mCh. A) and C) Data of single experiments of the maturation time versus lag-time. B) and D) Data of single experiments of the maturation time versus growth rate. D) Two different regimes A and B can be distinguished and are separated by the red line for better visualization.

As a next step, we analyzed the maturation time of GFP for the R and C strain. As expected, the average GFP maturation time of the R strain was comparable to the S strain's maturation time with 5.48±0.4 min at similar growth rates (Table S2). GFP maturation in the C strain took place within 5.13±0.5 min at a slower average growth rate of 0.57±0.08 1/h and an increased lag-time of 64.3±10 min (Table S2). Although average differences in maturation times of GFP for the three strains are within measurement uncertainties (Figure S4), the analysis of MTs of single experiments showed a high variation in these maturation times (Figure 2). Maturation times in single experiments for the GFP expressing R and S strain, ranged from 5 to 6.2 min, with a weak dependency on the experimentally induced high variance in lag-times or growth rates (Figure 2A,B). In contrast, the C strain showed faster GFP maturation at increased lag-times or reduced growth rates with about 4 min at low growth rates as compared to 6 min at high growth rates. In general, a high variance in maturation times could be observed for all three strains.

### Different strains exhibit different GFP maturation profiles

The three strains additionally expressing GFP revealed a high variance in maturation times. Interestingly, the strains differed in their maturation profiles; the correlations between the maturation time and growth rate, lag-time or the maximal fluorescence intensity after CAP addition (as described below). To quantify these correlations we applied linear fits to the experimental data and determined the Pearson's correlation coefficient to obtain information on the correlation strength. The fit parameter and Pearons's correlation coefficients of Figure 2 are summarized in Table S4. For the S and R strain a weak positive correlation between MT and lag-time or growth rate was observed (Figure 2A,B), indicating that maturation of GFP in the S and R strain is not affected by growth conditions. A different result was obtained for the C strain. Here, a strong correlation between maturation time and growth conditions was present (Figure 2A,B). Maturation times were thereby negatively correlated with lag-time and positively correlated with growth rate. This was not surprising since C strain populations with a long lag-time in a particular experiment also grew slowly as soon as the population had left the lag-phase. We further observed that GFP maturation was faster in slow growing C strain populations. This finding was quite unexpected because growth conditions, as represented by the growth rate, should not have an effect on the maturation time, as growth ceases upon the addition of the antibiotic chloramphenicol (CAP). Since correlations of MTs with lag-time or growth rate were only weak for the R and S strain, we can rule out a direct dependency between growth conditions and maturation time. Our data rather indicate a C strain specific effect. In contrast to the S and R strain, the C strain possesses an additional plasmid that encodes the toxin Colicin. Replication as well as expression of this plasmid bears some metabolic costs leading to the significantly reduced growth rate (Table S1) and the prolonged lag-time of the C strain. As a consequence, the C strain has a different metabolism than the S and R strain that could affect the available oxygen concentration within a C strain bacterium. Therefore, our current hypothesis is that a slow growing C strain, due to its altered metabolism, has a higher oxygen availability at low growth rates that leads to the observed decrease of the maturation time with decreasing growth rate.

### Maturation profiles for mCherry

Whereas GFP maturation is well investigated both experimentally and theoretically [13], [23]–[27] (see Supporting Information S1 for details), maturation kinetics of mCherry is not equally well understood. Opposed to the exponential maturation kinetics of GFP, mCherry shows sigmoidal maturation kinetics (Figure 1C). After an initial lag-period with subsequent accelerated increase in fluorescence intensity (FI), we find an inflection point, decelerated increase and final saturation of FI. Kinetic studies of the maturation process of DsRed, an ancestor of mCherry [25], suggested that the DsRed maturation process can theoretically be formulated as a three-step process [25]. Here, we analyzed the maturation kinetics of the fluorescent protein mCherry. As a derivative of DsRed [18], mCherry chromophore formation has to undergo two subsequent oxidation steps [29]. The first oxidation step creates a green intermediate [28], and the second oxidation step leads to an increase in conjugated double bonds. As a consequence, one observes a shift to longer absorbance and emission wavelengths [28]. We measured both the temporal fluorescence development of the green intermediate and of the red fluorescent state of the final mCherry protein in parallel (Figure S5). We obtained the characteristic sigmoidal fluorescence development for the red fluorescent protein after CAP addition. Interestingly, fluorescence intensities of the green intermediate saturated at a later point in time compared to the FI of the red fluorescent protein, although the red fluorescent state is built up from the green fluorescent state. Another characteristic of the FI profile of mCherry is a quasi-linear regime prior to saturation (Figure S5).

In Supporting Information S1, we present a theoretical model based on a two-step process that includes a catalytic back-reaction from the red fluorescent state to the green fluorescent state. This nonlinear reaction model explains our experimental data in very good agreement (Figure S5) and justifies the application of a sigmoidal fit function for the measurement of the mCherry maturation time (see Material and Methods, Figure 1C). We observed that mCherry expressed by the S strain matured in 70.3±11.7 min and thus on a significantly longer time-scale than GFP maturation. The longer MT can be attributed to the additional oxidation step that is necessary for chromophore formation [29]. Whereas mCherry expressed by the R strain matured in 77.7±8.5 min and hence on a time scale similar to that of the S strain, mCherry matured significantly faster in the slow growing C strain with 59.4±7.5 min. Again, a high variability in maturation times for all three strains was observed.

Similar to the maturation profiles of GFP a weak positive correlation between mCherry maturation and lag-time could be observed for the S and R strain, while maturation of mCherry in the C strain was negatively correlated (Figure 2C). In contrast, maturation times of mCherry were strongly positively correlated with growth rate for all three strains (Figure 2D), with two different regimes A and B that could be distinguished: Regime A includes experiments at small growth rates ranging from 0.3 to 0.65 1/h, regime B represents experiments at high growth rates ranging from 0.6 to 0.85 1/h. For both regimes, a similar dependence of MTs on growth rate was present, as strains in experiments with higher growth rates showed longer maturation times. Notably, at the transition from regime A to B, maturation times dropped abruptly for all strains. The C strain showed the lowest growth rate in both regimes and matured the fastest with 67±10 min in regime A (compared to 79±14 min (S) and 91±11 min (R)) and 52±5 min in regime B (compared to 61±9 min (S) and 64±6 min (R)) (Figure 2D, Table S5).

### Strains expressing additional growth rate reducing plasmids show different maturation behavior

Unexpectedly, the maturation times of mCherry expressed by all three strains are strongly correlated with growth rate, which was not the case for GFP. For GFP, this dependency could only be observed for the C strain. A closer look at Figure 2B and Figure 2D reveals that strains expressing mCherry in Regime A are in the same growth rate range as the C strain expressing GFP. Both the GFP expressing C strain as well as the S, R, and C strain expressing mCherry carry additional plasmids, which lead to a significant growth rate reduction. Whereas for the GFP expressing C strain this is the plasmid encoding the toxin Colicin, the growth rate reduction for the mCherry expressing strains results from the mCherry expression itself. The plasmid expressing GFP did not result in a significant growth rate reduction (Table S1). These growth rate reducing plasmids represent additional metabolic costs for the bacterial cell, and can lead to an altered metabolism, which in turn can affect the oxygen availability within the cell at the time-point of CAP addition and thereby maturation. As a consequence, these above described slow growing bacterial populations could have higher oxygen concentrations within the cell allowing for faster maturation.

### GFP versus mCherry maturation

**Figure 3:**
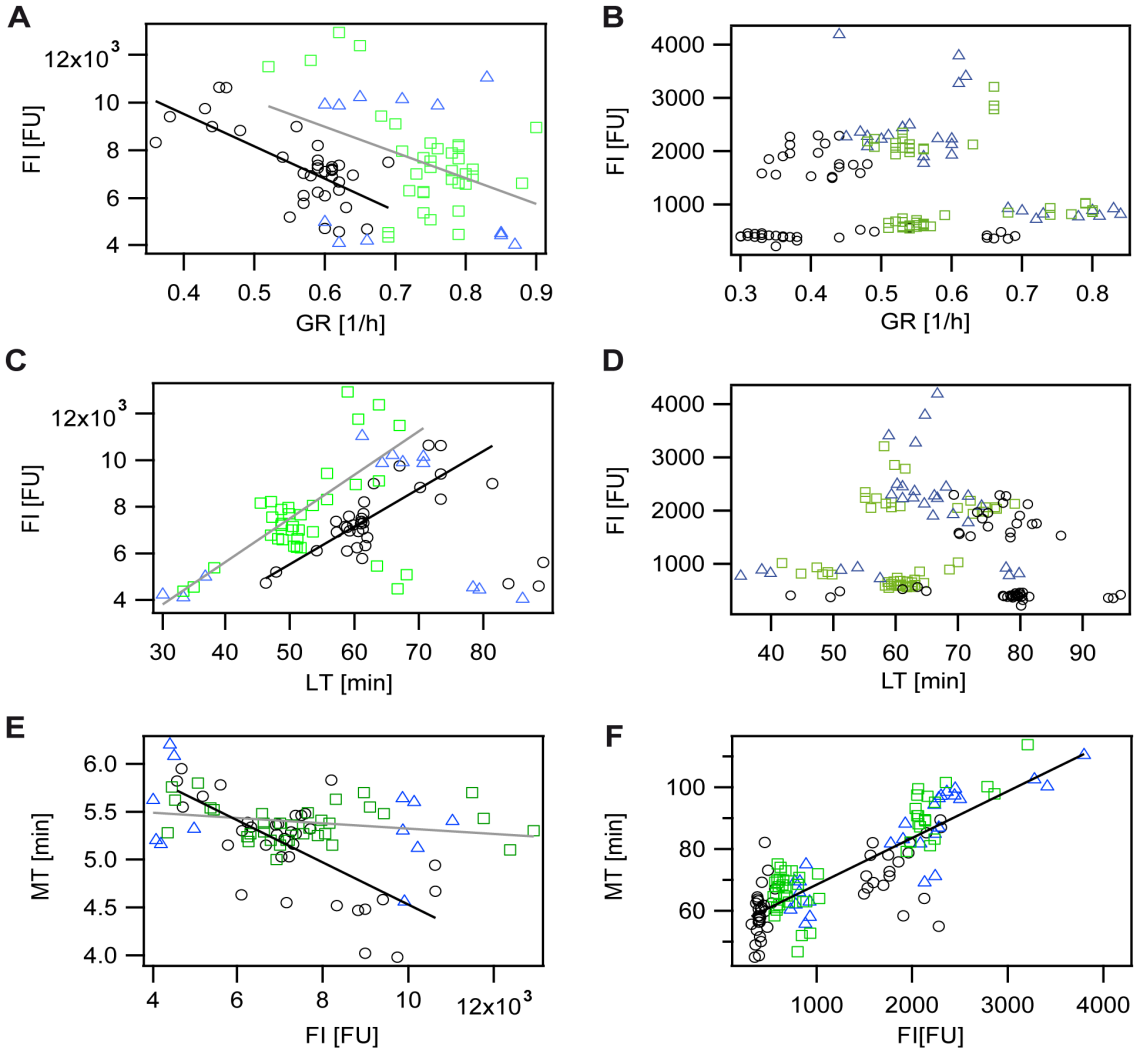
Analysis of factors influencing maturation time (MT) of fluorescence proteins expressed in the S (green rectangles), R (blue triangles), and C strain (black circles). Solid lines: fits to experimental data. Fit-parameter and Pearson's correlation coefficients can be found in Table S6. A), B) Fluorescence intensity (FI) versus growth rate (GR). C), D) Fluorescence intensity versus lag-time (LT). E), F) Maturation time versus maximal fluorescence intensity after CAP addition. A), C), E) strains expressing GFP. B), D), F) strains expressing mCherry.

In order to understand the maturation behavior of FPs in our strains in more detail, we performed additional correlation studies (Figure 3). The according fit parameter and Pearson's correlation coefficients are given in Table S6. For GFP expressing strains a negative correlation of maximal fluorescence intensity (after CAP addition) with growth rate and a positive correlation of maximal FI with lag-time were observed. As seen for the correlations of the MT with growth conditions, the dependencies were more pronounced for the C strain in comparison to the S and R strain. Analyzing the relation between the MT and the maximal FI determined a weak negative correlation for the S and R strain as opposed to a strong negative correlation for the C strain expressing GFP. Taken all data given in Figure 2A,B and Figure 3A,C,E together, our data show that in slow growing C cells more fluorescent protein is present at the time point of CAP addition, and that slow growing C cells are able to mature GFP in less time than fast growing ones. It was previously shown [12] that the amount of unnecessarily expressed proteins such as β-galactosidase (in our case the FP GFP) can be correlated with growth rate. Nevertheless, it is puzzling that slow growing GFP expressing C cells are able to mature the high amount of GFPs in less time. One possible explanation could be that due to the different metabolism present in fast versus slow growing *E. coli* cells [46], the C cells are able to mature GFP faster under slow growth conditions. We hypothesize that the altered metabolism could affect the oxygen availability within the bacterial cells, and thereby allows the C strain to mature even high fluorescent protein concentrations in less time.

For mCherry, no correlations between the maximal fluorescence intensity (after CAP addition) with growth rate or lag-time could be observed. Instead, independent of the strain studied, two different amounts of the FP were produced (clouds at 750 FI or 2000 FI, see Figure 3F). The production of the fluorescent protein mCherry is therefore not dependent on growth conditions. Nevertheless, a strong positive correlation between the maturation time of mCherry expressed by all three different strains and the maximal fluorescence intensity (after CAP addition) was determined (Figure 3F). As mentioned above, mCherry maturation requires two subsequent oxidation steps in contrast to only one for GFP maturation. Therefore, mCherry maturation seems to be strongly dependent on oxygen availability. At high mCherry concentrations oxygen could become limiting, thereby leading to the observed dependency of maximal FI reached after CAP addition on mCherry maturation time.

## Discussion

In this study we explored the maturation of the fluorescent proteins GFPmut3 and mCherry additionally expressed by the three strains of the *Escherichia coli* Colicin E2 system, a model system for colicinogenic interaction. In this model system, the strains' genetic differences are coupled to differences in growth rate [6]. This setup allowed us to address the question whether maturation behavior differs even between closely related strains. In such a case, the differences in maturation kinetics of FPs between strains could bias the quantitative analysis of the strains' interactions or even change them qualitatively. Therefore, a thorough understanding of maturation is of high relevance, especially for the quantitative analysis of gene expression experiments. We experimentally induced variations in growth conditions [43] to study the maturation behavior of these strains in dependence on growth rate and lag-time. We aimed at investigating whether differences in growth conditions can affect maturation times of FPs and whether the maturation behavior is different for the two fluorescent proteins.

Whereas the R and S strain exhibit similar growth rates under the experimental conditions used in this study, the growth rate of the C strain is significantly slower. The average GFP maturation time of the three strains was 5.33 min with a high deviation ranging from 3.98 to 6.23 min and was comparable to previously reported MTs for this fluorescent protein [13], [38]. GFP fluorescence development after CAP addition followed the expected exponential shape as opposed to the sigmoidal FI development of the mCherry fluorescence. Whereas maturation kinetics of the mCherry ancestor DsRed was theoretically described by a three-step process [25], our experimentally observed data for mCherry maturation kinetic was adequately described by a two-step process with an additional back reaction from the red fluorescent state to the green fluorescent state (Supporting Information S1). The average mCherry maturation time of the S, R and C strain was 69 min, with a high variation ranging from 45 to 114 min. Our three *E. coli* strains showed significantly longer mCherry maturation times than for example *E. coli* LMG194 with 15 min [18] or PAmCherrys expressed in *E. coli* LMG194 with 18 to 25 min [19].

Our data show that maturation times of FPs are highly variable and differ for the three closely related strains used in this study. Whereas for the GFP expressing C strain maturation times are strongly correlated with growth conditions (represented by the parameters lag-time and growth rate), these correlations are considerably weaker for the S and R strain. Doubling the growth rate of the GFP expressing C strain results in an increased maturation time of GFP by a factor of 1.4, but only of 1.1 in the S and R strains.

Although at first thought a dependency of maturation times on growth rate seems contradictory, it can be explained by different metabolisms being present in the studied strains. For the GFP expressing C strain, the additional replication or expression of the toxin encoding plasmid leads to a significant growth rate reduction that can affect the C strain's metabolism. A similar coherence can be present for the S, R, and C strains expressing mCherry. Again, the introduction of mCherry expressing plasmids leads to a significant growth rate reduction (Table S1) that might interfere with the cells' metabolism. As a consequence, oxygen availability can vary in these strains and explain the observed differences in maturation times and maturation behavior.

Fluorescent proteins are widely used to analyze kinetics of protein expression. The recorded data are often directly applied in theoretical modeling, resulting in predictions on quantitative gene expression of a bacterial population. Caution has to be excercised when comparing gene expression profiles of various bacterial mutants of the same species since their maturation profile can differ significantly as is highlighted in this study. These variations of maturaton times become particularly important when gene expression is studied on a single cell level. In this case, cell-to-cell variations in growth rates, which reflect differences in metabolism could lead to variations in maturation times of the fluorescent protein that is used to quantify gene expression. This dependency has to be accounted for in the theoretical modeling to thoroughly infer the dynamics of the protein of interest. Therefore, experimental determination of growth rate as well as maturation time distributions [38] could reduce discrepancies between experimental, quantitative data obtained using single cell fluorescence time-lapse microscopy [47] and theoretical results. Increasing understanding of maturation dynamics of FPs as well as their affecting factors, will allow us to broaden the applicability of fluorescent proteins as gene expression markers on a single cell level or simply to analyze bacterial population dynamics.

## Supporting Information

Figure S1 **Normalized growth curves of S (black rectangles), R (grey triangles), and C strain (blue circles) in liquid M63 medium.** A) no fluorescent protein expressed (nfp), B) green fluorescent protein expressed (GFP), C) red fluorescent protein expressed (mCh). Growth rates (GR) are given in Table S1. Averages are taken over a typical data set of three replicas.
(TIF)

Figure S2 **Effect of different CAP concentrations on maturation times of the FPs GFP and mCherry expressed in the S, R and C strain.** To address the question whether the translation inhibition by the antibiotic chloramphenicol (CAP) might be different for the three strains, we performed an additional set of experiments in which we compared the maturation times of the FPs GFP and mCherry expressed by the three strains S, R, and C after addition of 100 µg/ml (black) or 200 µg/ml (blue) CAP, respectively (Table S3). In both data sets comparable maturation times were observed. This is in agreement with the previously obtained data presented in Figure 2. Therefore, we can rule out that the differences of maturation times between the S, R, and C strain might be due to ineffective translation inhibition by the antibiotic chloramphenicol in these strains. A) Maturation time (MT) of the FP GFP for the S, R, and C strain. B) Maturation time of the FP mCherry for the S, R, and C strain.
(TIF)

Figure S3 **Stability of the FPs GFP and mCherry in the S, R, and C strain.** As stated in the literature [18], [44], [45], GFPmut3 and mCherry are very stable fluorescent proteins. The half-life time of GFPmut3 has been shown to be 24 hours in *E. coli* [45]. To rule out that these FPs might be less stable in the three specific strains used in this study, we monitored the fluorescence intensity after CAP addition for several hours and found both FPs to be stable in all three strains. Data are given from the time point of CAP addition. A) Normalized fluorescence intensity of the FP GFP in the S (black), R (grey), and C (blue) strain. B) Normalized fluorescence intensity of the FP mCherry in the S (black), R (grey), and C (blue) strain. Since for the FP mCherry a slight decrease of the FI signal could be observed, only data points in the time frame 180 – 400 min have been used to accurately fit the data. (see Material and Methods).
(TIF)

Figure S4 **Growth, maturation, and fluorescence expression analysis for S, R, and C strain expressing the fluorescent proteins GFP and mCherry, respectively.** A),C),E),G) strains expressing GFP. B),D),F),H) strains expressing mCherry. A,B) Growth rate (GR). C),D) Lag-time (LT). E),F) Maturation time (MT). G),H) Fluorescence intensity (FI). Errors are given as the standard deviation σ.
(TIF)

Figure S5 **Theoretical analysis of GFP and mCherry maturation process in comparison to the normalized experimental data.** A) Fluorescence development of GFP expressed by the S strain after 200 µg/ml CAP addition at time T_0_ (arrow). Solid line: exponential fit as obtained from fitting the one-step model described in Supporting Information S1. B) Fluorescence development of mCherry expressed by the S strain. Fluorescence intensities of the green intermediate (grey circles) and the final red fluorescent protein (black crosses) are plotted versus time. Experimental data are given as averages of three typical data sets. The arrow indicates the time-point of CAP addition (200 µg/ml) after 180 min. C) Same data as shown in B), but beginning at the time-point of CAP addition T_0_ (arrow). Fluorescence intensity of the green intermediate (grey circles) and fluorescence development of the final red fluorescent protein (black crosses) are plotted versus time. Error bars represent the standard deviation. Solid lines: numerical solution as obtained from the theoretical analysis (see Supporting Information S1). The theoretical analysis reproduces the main experimental findings: the sigmoidal shape of the fluorescence development of the final fluorescent protein, the saturation of FI of the final red fluorescent state prior to the green fluorescent intermediate, and the quasi-linear regime of fluorescence development of the green fluorescent intermediate prior to saturation.
(TIF)

Table S1 **Growth rates of strains S, R, and C in liquid M63 medium. **
(DOCX)

Table S2 **Growth rates, lag-times and maturation times for S, R, and C strain expressing the fluorescent protein GFP at 200 µg/ml CAP.**
(DOCX)

Table S3 **Growth rates and maturation times for S, R, and C strain expressing the fluorescent proteins GFP or mCherry at 100 or 200 µg/ml CAP.**
(DOCX)

Table S4 **Parameters of fits applied to the data presented in** **Figure 2** **.**
(DOCX)

Table S5 **Growth rates, lag-times and maturation times for S, R, and C strain expressing the fluorescent protein mCherry at 200 µg/ml CAP.**
(DOCX)

Table S6 **Parameters of fits applied to the data presented in** **Figure 3** **.**
(DOCX)

Supporting Information S1 Description of the theoretical model for GFP and mCherry maturation.
(DOC)
