# Supplementary figures and images for: High Variation of Fluorescence Protein Maturation Times in Closely Related Escherichia coli Strains
Source: PLoS One. 2013 Oct 14;8(10):e75991. doi: 10.1371/journal.pone.0075991 (PMC3796512; doi:10.1371/journal.pone.0075991)

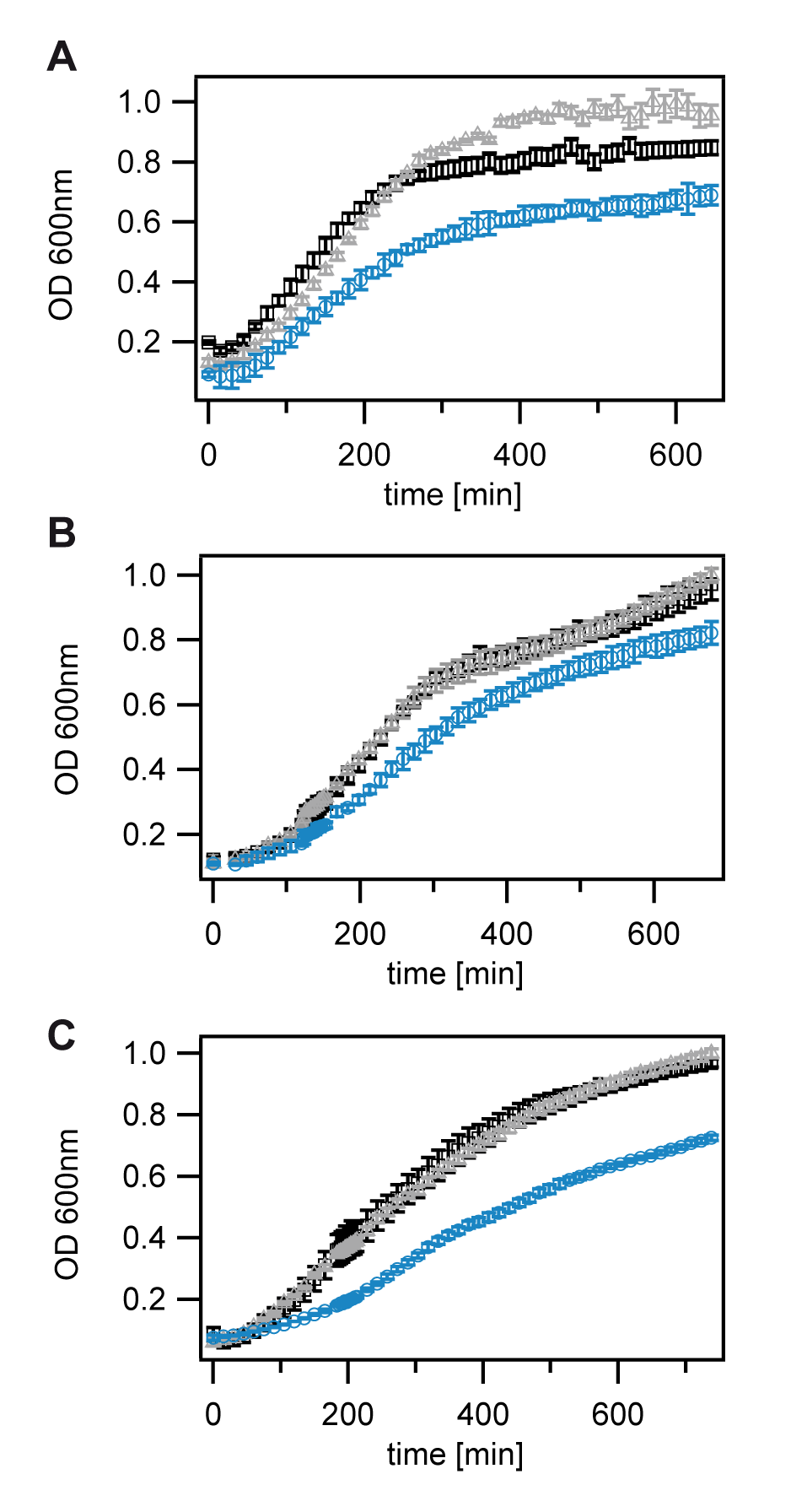

Supplement: Figure S1 — Normalized growth curves of S (black rectangles), R (grey triangles), and C strain (blue circles) in liquid M63 medium. A) no fluorescent protein expressed (nfp), B) green fluorescent protein expressed (GFP), C) red fluorescent protein expressed (mCh). Growth rates (GR) are given in Table S1. Averages are taken over a typical data set of three replicas. (TIF) [file pone.0075991.s001.tif]

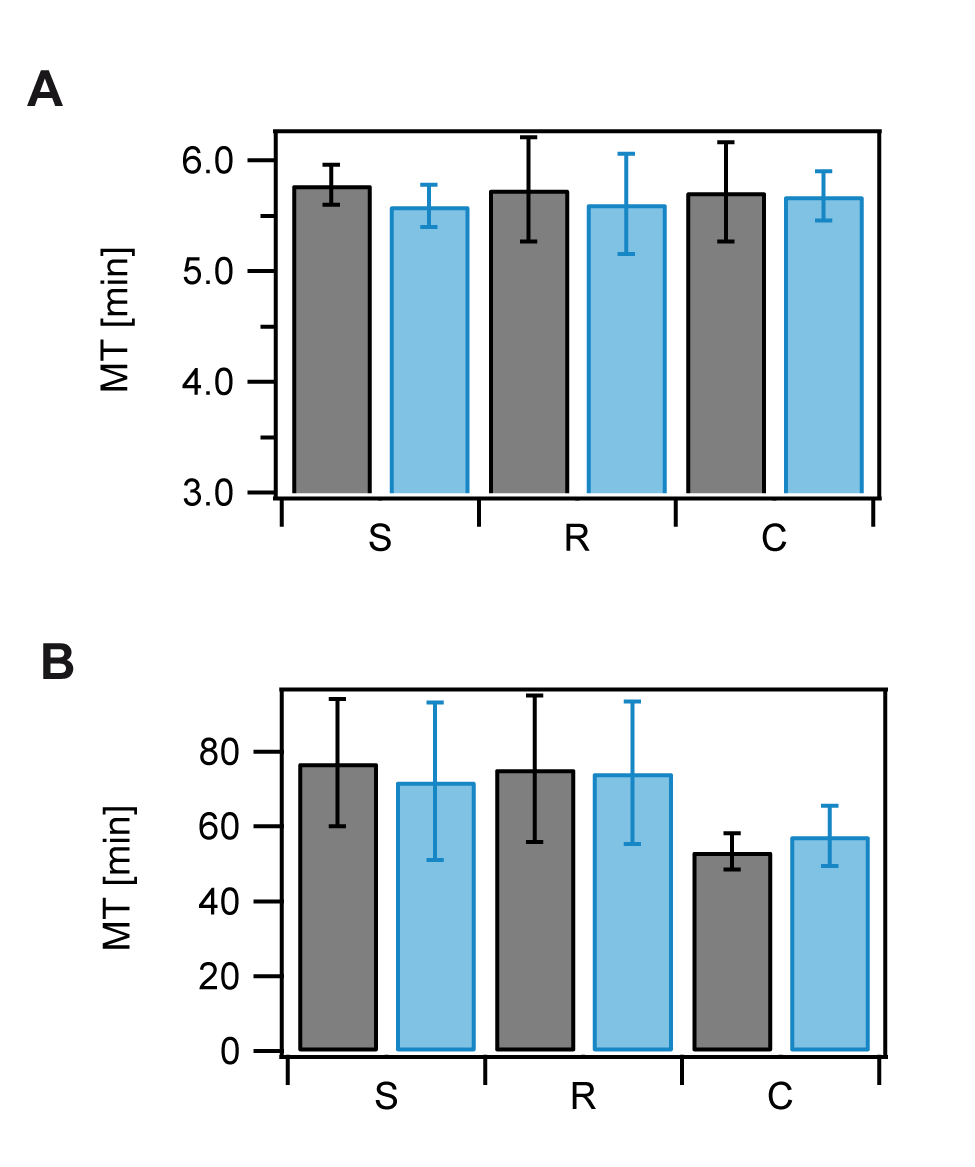

Supplement: Figure S2 — Effect of different CAP concentrations on maturation times of the FPs GFP and mCherry expressed in the S, R and C strain. To address the question whether the translation inhibition by the antibiotic chloramphenicol (CAP) might be different for the three strains, we performed an additional set of experiments in which we compared the maturation times of the FPs GFP and mCherry expressed by the three strains S, R, and C after addition of 100 µg/ml (black) or 200 µg/ml (blue) CAP, respectively (Table S3). In both data sets comparable maturation times were observed. This is in agreement with the previously obtained data presented in Figure 2. Therefore, we can rule out that the differences of maturation times between the S, R, and C strain might be due to ineffective translation inhibition by the antibiotic chloramphenicol in these strains. A) Maturation time (MT) of the FP GFP for the S, R, and C strain. B) Maturation time of the FP mCherry for the S, R, and C strain. (TIF) [file pone.0075991.s002.tif]

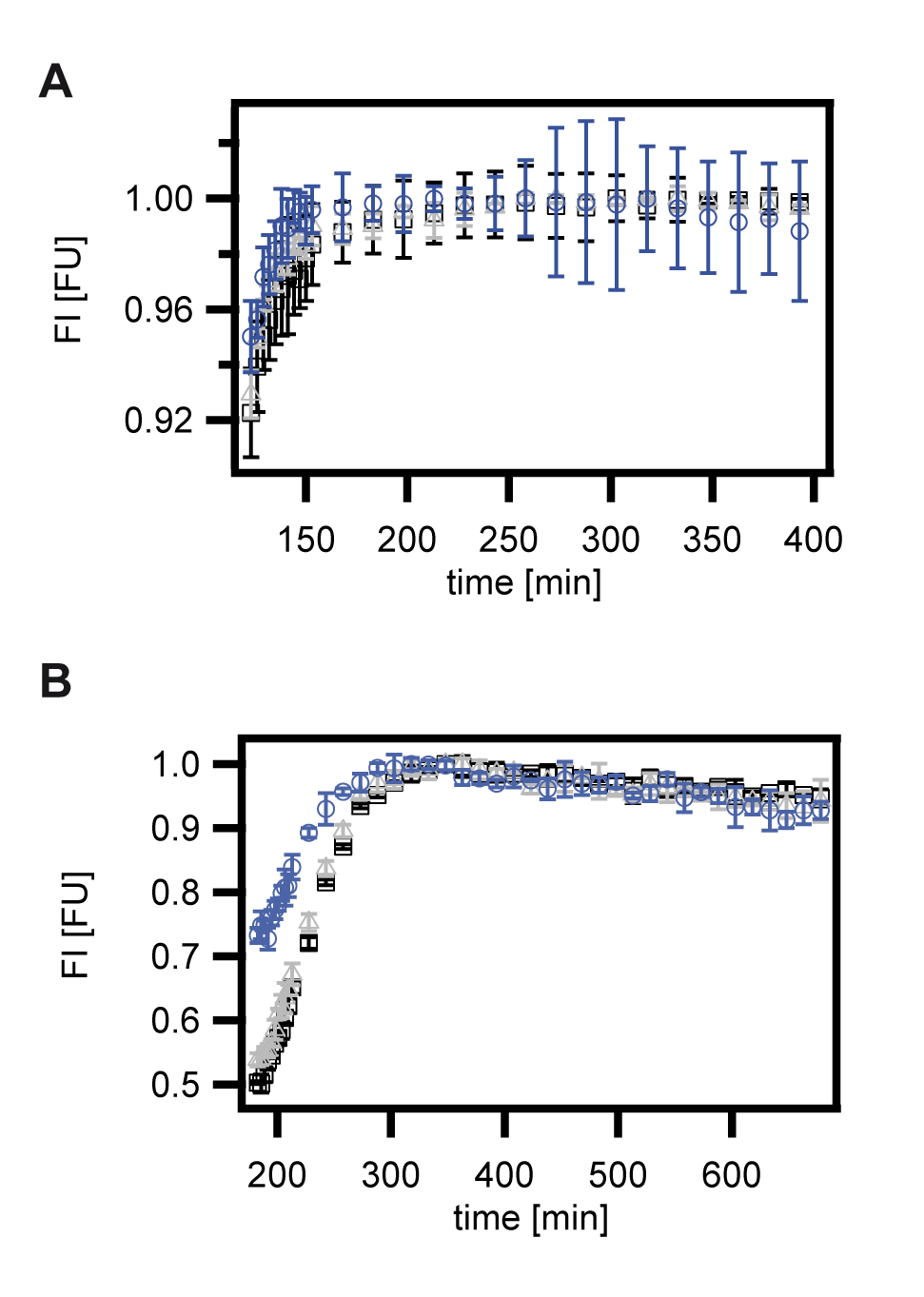

Supplement: Figure S3 — Stability of the FPs GFP and mCherry in the S, R, and C strain. As stated in the literature [18], [44], [45], GFPmut3 and mCherry are very stable fluorescent proteins. The half-life time of GFPmut3 has been shown to be 24 hours in E. coli [45]. To rule out that these FPs might be less stable in the three specific strains used in this study, we monitored the fluorescence intensity after CAP addition for several hours and found both FPs to be stable in all three strains. Data are given from the time point of CAP addition. A) Normalized fluorescence intensity of the FP GFP in the S (black), R (grey), and C (blue) strain. B) Normalized fluorescence intensity of the FP mCherry in the S (black), R (grey), and C (blue) strain. Since for the FP mCherry a slight decrease of the FI signal could be observed, only data points in the time frame 180 – 400 min have been used to accurately fit the data. (see Material and Methods). (TIF) [file pone.0075991.s003.tif]

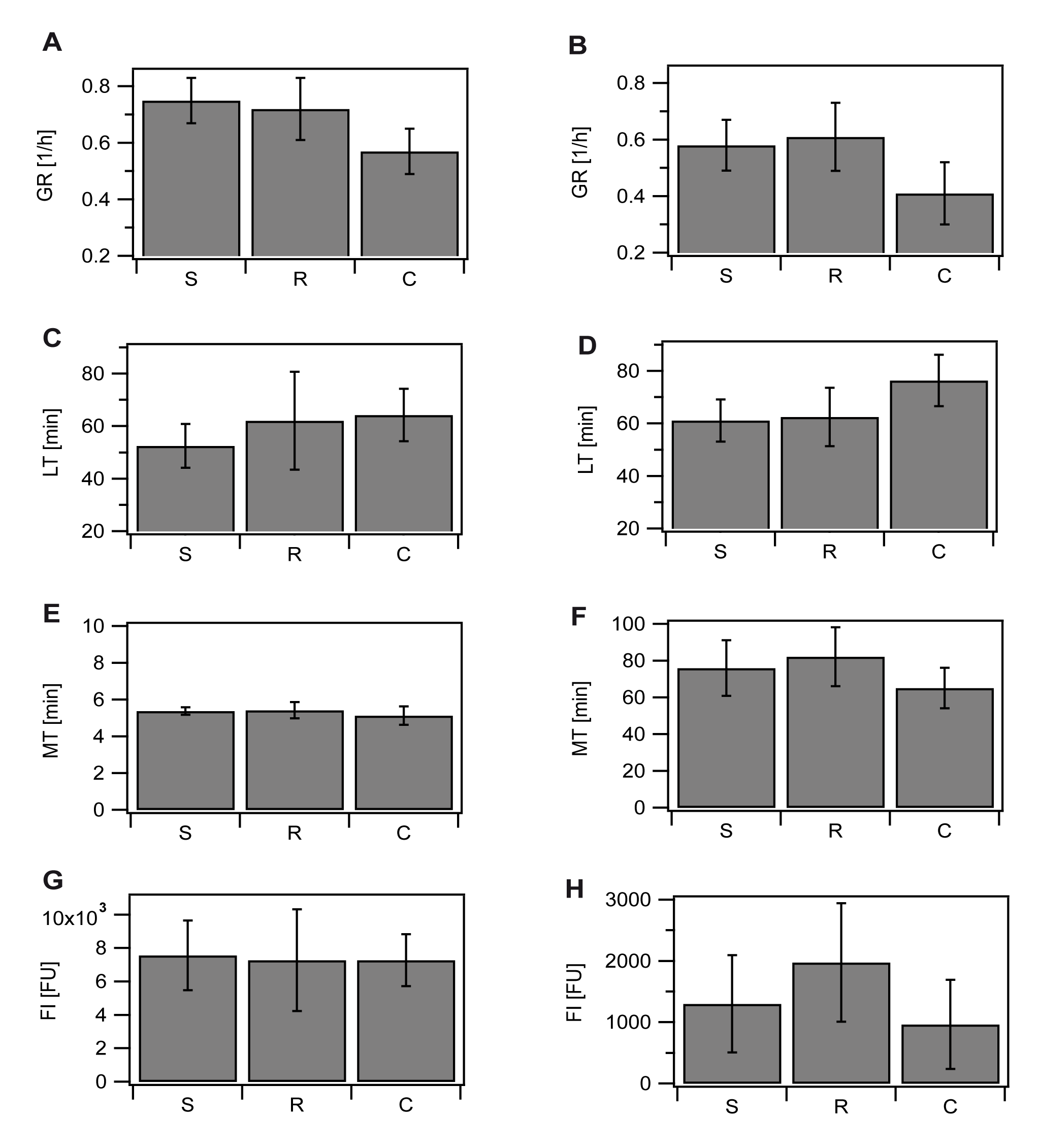

Supplement: Figure S4 — Growth, maturation, and fluorescence expression analysis for S, R, and C strain expressing the fluorescent proteins GFP and mCherry, respectively. A),C),E),G) strains expressing GFP. B),D),F),H) strains expressing mCherry. A,B) Growth rate (GR). C),D) Lag-time (LT). E),F) Maturation time (MT). G),H) Fluorescence intensity (FI). Errors are given as the standard deviation σ. (TIF) [file pone.0075991.s004.tif]

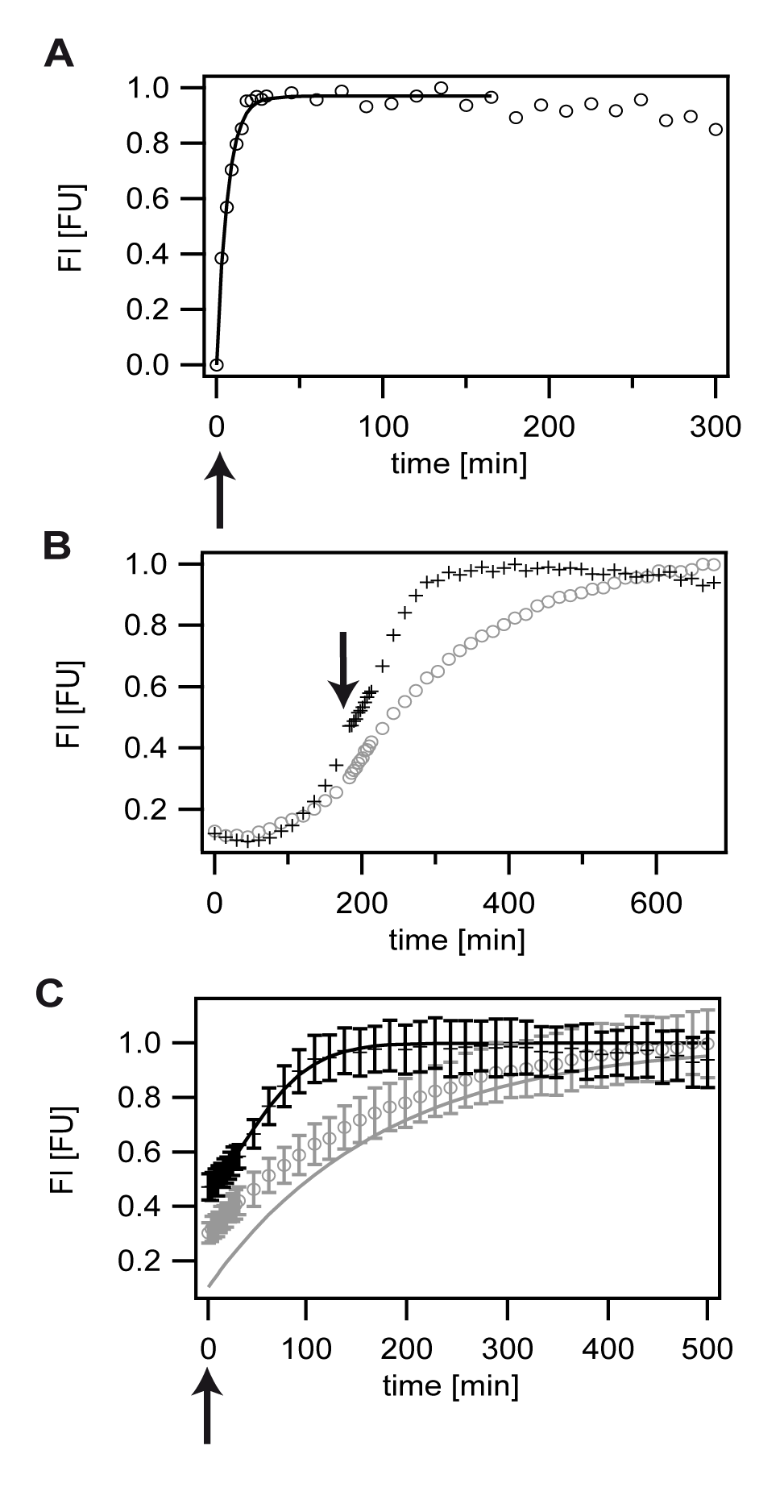

Supplement: Figure S5 — Theoretical analysis of GFP and mCherry maturation process in comparison to the normalized experimental data. A) Fluorescence development of GFP expressed by the S strain after 200 µg/ml CAP addition at time T0 (arrow). Solid line: exponential fit as obtained from fitting the one-step model described in Supporting Information S1. B) Fluorescence development of mCherry expressed by the S strain. Fluorescence intensities of the green intermediate (grey circles) and the final red fluorescent protein (black crosses) are plotted versus time. Experimental data are given as averages of three typical data sets. The arrow indicates the time-point of CAP addition (200 µg/ml) after 180 min. C) Same data as shown in B), but beginning at the time-point of CAP addition T0 (arrow). Fluorescence intensity of the green intermediate (grey circles) and fluorescence development of the final red fluorescent protein (black crosses) are plotted versus time. Error bars represent the standard deviation. Solid lines: numerical solution as obtained from the theoretical analysis (see Supporting Information S1). The theoretical analysis reproduces the main experimental findings: the sigmoidal shape of the fluorescence development of the final fluorescent protein, the saturation of FI of the final red fluorescent state prior to the green fluorescent intermediate, and the quasi-linear regime of fluorescence development of the green fluorescent intermediate prior to saturation. (TIF) [file pone.0075991.s005.tif]
