# Supplementary material for: High Variation of Fluorescence Protein Maturation Times in Closely Related Escherichia coli Strains
Source: PLoS One. 2013 Oct 14;8(10):e75991. doi: 10.1371/journal.pone.0075991 (PMC3796512; doi:10.1371/journal.pone.0075991)
Supplement: Table S1 — Growth rates of strains S, R, and C in liquid M63 medium. (DOCX) [file pone.0075991.s006.docx]

**Table S1:** **Growth rates of strains S, R, and C in liquid M63 medium.**

| **Strain** | **Nfp** | **GFP** | **mCh** |
| --- | --- | --- | --- |
| S | 0.74 ± 0.03 | 0.73 ± 0.03 | 0.63 ± 0.03 |
| R | 0.80 ± 0.02 | 0.75 ± 0.04 | 0.58 ± 0.03 |
| C | 0.63 ± 0.04 | 0.55 ± 0.01 | 0.45 ± 0.01 |

Growth rates have been obtained by using a microplate-reader as described in the materials and methods section. Growth rates are given for strains not expressing a fluorescent protein (nfp), for strains expressing green fluorescent protein (GFP), as well as for strains expressing mCherry (mCh). Growth rate (GR) is given in [1/h] with standard deviation σ. Growth rates given here correspond to the data sets shown in Figure S1.
