# Supplementary material for: High Variation of Fluorescence Protein Maturation Times in Closely Related Escherichia coli Strains
Source: PLoS One. 2013 Oct 14;8(10):e75991. doi: 10.1371/journal.pone.0075991 (PMC3796512; doi:10.1371/journal.pone.0075991)
Supplement: Table S2 — Growth rates, lag-times and maturation times for S, R, and C strain expressing the fluorescent protein GFP at 200 µg/ml CAP. (DOCX) [file pone.0075991.s007.docx]

**Table S2: Growth rates, lag-times and maturation times for S, R, and C strain expressing the fluorescent protein GFP at 200 μg/ml CAP.**

| **Strains** | **GFP** | | |
| --- | --- | --- | --- |
|  | **GR [1/h]** | **LT [min]** | **MT [min]** |
| **S** | 0.75 ± 0.08 | 52.5 ± 8.3 | 5.38 ± 0.20 |
| **R** | 0.72 ± 0.11 | 62.1 ± 18.6 | 5.48 ± 0.43 |
| **C** | 0.57 ± 0.08 | 64.3 ± 10.0 | 5.13 ± 0.50 |

Growth rate (GR) is given in [1/h] with standard deviation σ. Lag-time (LT) is given in [min] with standard deviation σ. Maturation time (MT) is given in [min] with standard deviation σ.
