# Supplementary material for: High Variation of Fluorescence Protein Maturation Times in Closely Related Escherichia coli Strains
Source: PLoS One. 2013 Oct 14;8(10):e75991. doi: 10.1371/journal.pone.0075991 (PMC3796512; doi:10.1371/journal.pone.0075991)
Supplement: Table S3 — Growth rates and maturation times for S, R, and C strain expressing the fluorescent proteins GFP or mCherry at 100 or 200 µg/ml CAP. (DOCX) [file pone.0075991.s008.docx]

**Table S3: Growth rates and maturation times for S, R, and C strain expressing the fluorescent proteins GFP or mCherry at 100 or 200 μg/ml CAP.**

| **Strains** | **GFP**  **(100 μg/ml CAP)** | | **GFP**  **(200 μg/ml CAP)** | |
| --- | --- | --- | --- | --- |
|  | **GR [1/h]** | **MT [min]** | **GR [1/h]** | **MT [min]** |
| **S** | 0.69 ± 0.04 | 5.78 ± 0.18 | 0.74 ± 0.04 | 5.59 ± 0.19 |
| **R** | 0.70 ± 0.07 | 5.74 ± 0.47 | 0.74 ± 0.13 | 5.61 ± 0.45 |
| **C** | 0.56 ± 0.07 | 5.72 ± 0.45 | 0.61 ± 0.04 | 5.68 ± 0.22 |
|  | **mCh**  **(100 μg/ml CAP)** | | **mCh**  **(200 μg/ml CAP)** | |
|  | **GR [1/h]** | **MT [min]** | **GR [1/h]** | **MT [min]** |
| **S** | 0.71 ± 0.06 | 77.0 ± 17.0 | 0.72 ± 0.07 | 72.1 ± 21.0 |
| **R** | 0.69 ± 0.08 | 75.4 ± 19.6 | 0.73 ± 0.08 | 74.3 ± 19.0 |
| **C** | 0.55 ± 0.15 | 53.3 ± 4.8 | 0.55 ± 0.13 | 57.5 ± 8.1 |

This table summarizes the data obtained in Figure S2. Growth rate (GR) is given in [1/h] with standard deviation σ. Maturation time (MT) is given in [min] with standard deviation σ. Please note that the C strain expressing GFP in these experiments has been growing relatively fast, and therefore needs the same time to mature the FP GFP as the S and R strains. Strains expressing the FP mCherry were in the B regime as can be seen in Figure 2D. In summary, differences in MT between measurements with 100 and 200 μg/ml CAP are within the error range and are in accordance with the previously obtained data. We can therefore rule out that the differences of maturation times between the S, R, and C strain might be due to ineffective translation inhibition by the antibiotic chloramphenicol (CAP). Experiments were performed on two separate days, with three replicas each.
