# Supplementary material for: High Variation of Fluorescence Protein Maturation Times in Closely Related Escherichia coli Strains
Source: PLoS One. 2013 Oct 14;8(10):e75991. doi: 10.1371/journal.pone.0075991 (PMC3796512; doi:10.1371/journal.pone.0075991)
Supplement: Table S4 — Parameters of fits applied to the data presented in Figure 2 . (DOCX) [file pone.0075991.s009.docx]

**Table S4: Parameters of fits applied to the data presented in Figure 2.**

| **Fit** | **Specification** | **Parameter**  **A** | **Parameter**  **B** | **Excluded**  **data points** | **Pearson’s**  **correlation coefficient r** |
| --- | --- | --- | --- | --- | --- |
| **Fig. 2a black line** | C strain | 7.85 ± 0.5 | -0.05 ± 0.0 | 3 (at LT > 90 min) | -0.7 |
| **Fig. 2a grey line** | S + R strain combined | 4.85 ± 0.2 | 0.01 ± 0.0 |  | 0.44 |
| **Fig. 2b black line** | C strain | 2.61 ± 0.5 | 4.47 ± 0.8 |  | 0.71 |
| **Fig. 2b grey line** | S + R strain combined | 4.74 ± 0.4 | 0.89 ± 0.5 |  | 0.27 |
| **Fig. 2c black line** | C strain | 129.4 ± 17 | -0.81 ± 0.2 | 3 (at LT < 55 min) | -0.5 |
| **Fig. 2c grey line** | S + R strain combined | 56.8 ± 12 | 0.35 ± 0.2 |  | 0.2 |
| **Fig. 2d black line** | All strains combined Regime A | 33.4 ± 6.4 | 89.5 ± 13.2 |  | 0.57 |
| **Fig. 2d grey line** | All strains combined Regime B | -10.6 ± 12.5 | 96.5 ± 17 |  | 0.77 |

Given are the parameters of the linear fits (y = b·x + a) applied to the data of Figure 2. The Pearson’s correlation coefficient r reveals the strength of the correlation with -1 < r < 1. Values of r close to 0 indicate no correlation, values close to -1 or 1 indicate strong correlation. Figure 2A,B (grey rows): GFP, Figure 2C,D (white rows): mCh.
