# Supplementary material for: High Variation of Fluorescence Protein Maturation Times in Closely Related Escherichia coli Strains
Source: PLoS One. 2013 Oct 14;8(10):e75991. doi: 10.1371/journal.pone.0075991 (PMC3796512; doi:10.1371/journal.pone.0075991)
Supplement: Table S5 — Growth rates, lag-times and maturation times for S, R, and C strain expressing the fluorescent protein mCherry at 200 µg/ml CAP. (DOCX) [file pone.0075991.s010.docx]

| **Strains** | **mCh** | | | **mCh (A)** | | **mCh (B)** | |
| --- | --- | --- | --- | --- | --- | --- | --- |
|  | **GR [1/h]** | **LT [min]** | **MT [min]** | **GR [1/h]** | **MT [min]** | **GR [1/h]** | **MT [min]** |
| **S** | 0.58 ± 0.09 | 61.2 ± 8.0 | 70.33 ± 11.7 | 0.55 ± 0.04 | 79.23 ± 14.4 | 0.74 ± 0.07 | 61.43 ± 9.0 |
| **R** | 0.61 ± 0.12 | 62.5 ± 11.1 | 77.69 ± 8.5 | 0.55 ± 0.05 | 91.08 ± 10.9 | 0.77 ± 0.06 | 64.29 ± 6.1 |
| **C** | 0 42 ± 0.11 | 76.4 ± 0.11 | 59.37 ± 7.5 | 0.39 ± 0.06 | 67.01 ± 10.3 | 0.67 ± 0.01 | 51.72 ± 4.6 |

**Table S5: Growth rates, lag-times and maturation times for S, R, and C strain expressing the fluorescent protein mCherry at 200 μg/ml CAP.**

Growth rate (GR) is given in [1/h] with standard deviation σ. Lag-time (LT) is given in [min] with standard deviation σ. Maturation time (MT) is given in [min] with standard deviation σ. Since two different growth rate regimes (A and B, see main Figure 2D) can be observed for strains expressing the fluorescent protein mCherry, the data concerning growth rate and maturation time for these regimes are given in addition.
