# Supplementary material for: High Variation of Fluorescence Protein Maturation Times in Closely Related Escherichia coli Strains
Source: PLoS One. 2013 Oct 14;8(10):e75991. doi: 10.1371/journal.pone.0075991 (PMC3796512; doi:10.1371/journal.pone.0075991)
Supplement: Table S6 — Parameters of fits applied to the data presented in Figure 3 . (DOCX) [file pone.0075991.s011.docx]

**Table S6: Parameters of fits applied to the data presented in Figure 3.**

| **Fit** | **Specification** | **Parameter**  **A** | **Parameter**  **B** | **Excluded**  **data points** | **Pearson correlation coefficient r** |
| --- | --- | --- | --- | --- | --- |
| **Fig. 3a black line** | C strain | 14923 ± 1.4e^003^ | -13523 ± 2.5e^003^ |  | -0.69 |
| **Fig. 3a grey line** | S + R strain combined | 15419 ± 2.9e^003^ | -10726 ± 3.8e^003^ |  | -0.39 |
| **Fig. 3c black line** | C strain | -2553 ± 1.3e^003^ | 162 ± 21 | 3 (LT > 80 min) | 0.82 |
| **Fig. 3c grey line** | S + R strain combined | -1858 ± 935 | 187 ± 18 | 6 (LT > 60 at low FI) | 0.86 |
| **Fig. 3e black line** | C strain | 6.73 ± 0.3 | -0.0002 ± 4.15e^-005^ |  | -0.68 |
| **Fig. 3e grey line** | S + R strain combined | 5.6 ± 0.1 | -2.8e^-005^ ± 1.7e^-005^ |  | -0.24 |
| **Fig. 3f black line** | all strains combined | 53.4 ± 1.4 | 0.02 ± 0.0 |  | 0.84 |

Given are the parameters of the linear fits (y = b·x + a) applied to the data of Figure 3. The Pearson’s correlation coefficient r reveals the strength of the correlation with -1 < r < 1. Values of r close to 0 indicate no correlation, values close to -1 or 1 indicate strong correlation. Figure 3A,C,E (grey rows): GFP, Figure 3F (white row): mCh.
