# Supplementary material for: High Variation of Fluorescence Protein Maturation Times in Closely Related Escherichia coli Strains
Source: PLoS One. 2013 Oct 14;8(10):e75991. doi: 10.1371/journal.pone.0075991 (PMC3796512; doi:10.1371/journal.pone.0075991)
Supplement: Supporting Information S1 — Description of the theoretical model for GFP and mCherry maturation. (DOC) [file pone.0075991.s012.doc]

SUPPORTING INFORMATION for

**High variation of maturation times in closely related *Escherichia coli* strains**

Elke Hebisch^1^, Johannes Knebel^2^, Janek Landsberg^3^, Erwin Frey^2^ and Madeleine Leisner^2*^

^1^ Max-Planck-Institute for Biophysical Chemistry, Department of NanoBiophotonics, Am Fassberg 11, D-37077 Göttingen, Germany.

^2^ Arnold-Sommerfeld-Center for Theoretical Physics and Center for NanoScience, Department of Physics, Ludwig-Maximilians Universität München, Theresienstr. 37, 80333 München, Germany

^3^ Laboratoire Interdisciplinaire de Physique, Universite J. Fourier de Grenoble, 140, rue de la physique, 38402 Saint Martin d’Heres, France

* corresponding author: [Madeleine.Leisner@physik.uni-muenchen.de](mailto:Madeleine.Leisner@physik.uni-muenchen.de)

**Description of the theoretical model for GFP and mCherry maturation**

In the following we present a theoretical model describing the maturation kinetics of the fluorescent proteins GFP and mCherry.

*Maturation of GFP*

In accordance with previous studies (1-3), the maturation process of GFP is modeled as a one-step process from the immature, non-fluorescent state GFP^+^ to the green fluorescent state GFP^(G)^. An additional step in the transition scheme accounts for the degradation of the mature GFP^(G)^.

k_D_

[1]

k_G_

GFP^+^ GFP^(G)^ ∅

The reaction rates k_G_ and k_D_ determine the time scale of the transition to the fluorescent state and to the degraded state, respectively. The experimental data, however, suggest that the degradation of GFP^(G)^ takes place on a much longer time scale than the maturation process (k_D_ << k_G_) such that we may safely neglect the degradation process for the analysis of the maturation of GFP (Figure S5A).

The transition scheme [1] is best described by the following system of ordinary differential equations (ODEs),

$$\frac{d}{dt}[GFP^{+}](t)=-k_{G}\cdot[GFP^{+}](t),$$

[2]

$$\frac{d}{dt}[GFP^{(G)}](t)=k_{G}\cdot[GFP^{+}](t),$$

where [GFP^(i)^] denotes the concentration of the GFP compound with index i = +, G. From the ODE system [2] one obtains the solution for the concentration of the fluorescent state of GFP,

$$[GFP^{(G)}](t)=1-e^{-k_{G}\cdot t},$$

where the initial conditions are set to $[GFP^{+}](0)=1$ and $[GFP^{(G)}](0)=0$. The maturation time τ is the characteristic time scale of the exponential saturation process of GFP^(G)^ and is defined throughout this Supporting Material as the point in time at which the concentration of the mature compound under consideration reaches (1-1/e) ≈ 63% of its normalized maximal value. As a consequence, the maturation time of the fluorescent GFP^(G)^ state is computed as

[3]

$$\tau=\frac{1}{k_{G}}.$$

For the experimental data shown in Figure S5A we obtain the maturation time by fitting an exponential model of the form $[GFP^{(G)}](t)=A\cdot(1-e^{-k_{G}\cdot t})$ to the data. The outcome of this fit is presented in Theoretical Table T1.

**Theoretical Table T1: Fitting parameter and fit statistics of the exponential model for GFP maturation.** The fit statistics (t-statistic and P-value) strongly indicate the significance of the fit parameter k_G_ and A, and support the assumption of the exponential model for GFP maturation.

| **Fit parameter** | **Estimate**  **[min^-1^]** | **Standard error σ [min^-1^]** | **t-statistic** | **P-value** |
| --- | --- | --- | --- | --- |
| k_G_ | 0.153 | 0.006 | 25.79 | 1.2 ⋅10^-15^ |
| A | 0.970 | 0.007 | 141.35 | 7.2 ⋅10^-29^ |

The maturation time of GFP^(G)^ is obtained from this fit as

$$\tau_{GFP}=6.54\pm0.12\min.$$

In summary, our theoretical analysis confirms the assumption of a one-step process for the maturation of GFP. The extracted values for the maturation times from the experimental data are in line with previous measurements (4, 5).

*Maturation of mCherry*

The qualitative analysis of the data shown in Figure S5B,C suggests that the maturation of mCherry undergoes a multi-step process. As a derivative of DsRed (6), mCherry chromophore formation has to undergo two subsequent oxidation steps (7), as described in the main text. The first oxidation step thereby creates a green intermediate, whereas the second oxidation step results in the final red fluorescent state (8). The fluorescence development of both fluorescent states of mCherry was experimentally determined, at the same time. Interestingly, the shape of the red fluorescent saturation curve deviates from an exponential saturation and rather resembles a sigmoidal curve. In a previous study of DsRed (1), this experimental observation of the sigmoidal maturation kinetics was modeled as a three-step process. For the maturation of mCherry, however, we propose an extended two-step process with an additional catalyzed back-reaction, which can be summarized in the following reaction scheme,

k_G_

k_R_

[4]

mCherry^+^ mCherry^(G)^ mCherry^(R)^

k_B_

mCherry^(G)^ + mCherry^(G)^ mCherry^(R)^ + mCherry^(G)^

In this description, the maturation of mCherry undergoes a two-step process from the non-fluorescent state mCherry^+^ via the intermediate, green fluorescent mCherry^(G)^ to the mature, red fluorescent mCherry^(R)^. Thereby k_G_ and k_R_ denote the reaction rates of the respective transition. In addition, we assume a back-reaction from mCherry^(R)^ to mCherry^(G)^ with rate k_B_ which is mediated by the presence of mCherry^(G)^ itself. In this way mCherry^(G)^ acts as a catalyst for the back-reaction because the back-reaction depends on the concentration of mCherry^(G)^. In general, any chemical compound could trigger the back-reaction between the intermediate and the mature mCherry state. However, since it is not known in literature that additional proteins are involved in the maturation process of mCherry, we assume the intermediate green state of mCherry to catalyze the back-reaction from the mature mCherry^(R)^ to the intermediate mCherry^(G)^. The direct degradation of mCherry^(R)^ is neglected due to time-scale separation as is validated by our experimental data (Fig. S5B,C) and follows the same line of arguments as for the degradation of GFP.

The corresponding system of coupled ODEs for the introduced maturation process of mCherry [4] reads as follows,

$$\frac{d}{dt}[mCh^{+}](t)=-k_{G}\cdot[mCh^{+}](t),$$

[5]

$$\frac{d}{dt}[mCh^{(G)}](t)=k_{G}\cdot[mCh^{+}](t)-k_{R}\cdot[mCh^{(G)}](t)+k_{B}\cdot[mCh^{(G)}](t)\cdot[mCh^{(R)}](t),$$

$$\frac{d}{dt}[mCh^{(R)}](t)=k_{R}\cdot[mCh^{(G)}](t)-k_{B}\cdot[mCh^{(G)}](t)\cdot[mCh^{(R)}](t),$$

where [mCh^(i)^] denotes the concentration of the mCherry compound with index i = +, G, R.

In contrast to the transitions from mCherry^+^ to mCherry^(G)^ and from mCherry^(G)^ to the mature mCherry^(R)^, which translate to linear terms in the corresponding concentrations on the right-hand side of the ODE system [5], the catalytic back-reaction adds a nonlinear reaction term between the concentrations of mCherry^(G)^ and mCherry^(R)^ to the ODE system. Note that the differential equation for [mCh^(R)^] nearly matches the standard definition of a sigmoidal function if one replaces [mCh^(G)^] by [mCh^(R)^] in the last equation.

The sum over all concentrations of the three respective concentrations is conserved by the ODE system [5] and the nontrivial stationary solution is given by

$$[mCh^{+}]_{st}=0,$$

$$[mCh^{(G)}]_{st}=\frac{k_{B}-k_{R}}{k_{B}},$$

$$[mCh^{(R)}]_{st}=\frac{k_{R}}{k_{B}},$$

which enables us to normalize the concentrations to the interval [0,1].

By solving the ODE system [5] with the Runge-Kutta algorithm of 4^th^ order, implemented in Wolfram Mathematica 8.0, we empirically choose the following set of parameters and initial conditions to reproduce the experimental data depicted in Figure S5C. The range of the parameters was chosen in accordance with known values for other fluorescent proteins (2).

$$k_{G}=6.0\cdot10^{-3}\min^{-1},$$

$$k_{R}=2.0\cdot10^{-3}\min^{-1},$$

$$k_{B}=58.0\cdot10^{-3}\min^{-1},$$

$[mCh^{+}](0)=0.884,$ $[mCh^{(G)}](0)=0.100,$ $[mCh^{(R)}](0)=0.016$.

The outcome of this quantified reaction scheme is shown in Figure S5C as solid lines. The numerical solution of the ODE system [5] shows excellent agreement with the data for the red channel of mCherry^(R)^ and is in good agreement with the green channel of mCherry^(G)^. The systematic offset between the experimental data and the theoretical solution for the green mCherry^(G)^ channel might be assigned to measurement uncertainties in the maximal value of the mCherry^(G)^ concentration, which is propagated by data normalization.

The chosen reaction model reproduces the sigmoidal shape of the measured concentrations of mCherry^(R)^ and accounts for the fact that the green channel saturates on a longer time scale than the red channel. From a theoretical point of view, the latter feature strongly depends on the introduction of nonlinear reaction terms in the ODE system [5], which follows from the catalytic back-reaction together with the chosen reaction rates of the transitions and reactions. In our quantified reaction scheme, it is noteworthy that the catalytic back-reaction takes place on a much faster timescale than the transition from the intermediate to the mature mCherry state since k_B_ is much greater than k_R_.

The maturation time of mCherry^(R)^ is numerically determined from the solution of the ODE system [5] as

$$\tau_{mCh}=68.19\min,$$

which is in good agreement with the experimentally obtained value.

In summary, the theoretical considerations in comparison with the experimental data confirm our assumption of a two-step maturation process with a catalytic back-reaction from the red fluorescent state mCherry^(R)^ to the green fluorescent state mCherry^(G)^. Our results suggest that underlying catalytic reactions, which introduce nonlinearities to the corresponding ODE system, could be crucial for an understanding of the maturation kinetics of mCherry. Our theoretical analysis also justifies the use of a sigmoidal fit function to determine the maturation time of mCh.

**Supporting References**

1. Verkhusha VV, Akovbian NA, Efremenko EN, Varfolomeyev SD, Vryheshch PV (2001) Kinetic Analysis of Maturation and Denaturation of DsRed, a Coral-Derived Red Fluorescent Protein. Biochemistry (Moscow) 66: 1659-1670.

2. Reid BG, Flynn GC (1997) Chromophore Formation in Green Fluorescent Protein. Biochemistry 36: 6786-6791

3. Miyawaki A, Nagai R, Mizuno H (2003) Mechanisms of protein fluorophore formation and engineering. Curr Opin Chem Biol 7: 557-562.

4. Megerle JA, Fritz G, Gerland U, Jung K, Radler JO (2008) Timing and dynamics of single cell gene expression in the arabinose utilization system. Biophys J 95: 2103-2115.

5. Iizuka R, Yamagishi-Shirasaki M, Funatsu T (2011) Kinetic study of de novo chromophore maturation of fluorescent proteins. Anal Biochem. 414: 173-178.

6. Shaner NC, Campbell RE, Steinbach PA, Giepmans BNG, Palmer E (2004) Improved monomeric red, orange and yellow fluorescent proteins derived from *Discosoma sp.* red fluorescent protein. Nat Biotechnol 22: 1567-1572.

7. Shu X, Shaner NC, Yarbrough CA, Tsien RY, Remington SJ (2006) Novel Chromophores and Buried Charges Control Color in mFruits. Biochemistry 45: 9639 - 9647.

8. Matz MV, Arkady FF, Labas YA, Savitsky AP, Zaraisky AB, et al (1999) Fluorescent proteins from nonbioluminescent *Anthozoa* species. Nat Biotechnol 17: 969-973.
